# New-Onset Panic, Depression with Suicidal Thoughts, and Somatic Symptoms in a Patient with a History of Lyme Disease

**DOI:** 10.1155/2015/457947

**Published:** 2015-04-01

**Authors:** Amir Garakani, Andrew G. Mitton

**Affiliations:** ^1^Department of Psychiatry, Icahn School of Medicine at Mount Sinai, New York, NY 10029, USA; ^2^Silver Hill Hospital, New Canaan, CT 06840, USA; ^3^Department of Psychiatry, Yale School of Medicine, New Haven, CT 06511, USA; ^4^Department of Psychiatry, Semel Institute for Neuroscience and Human Behavior, University of California Los Angeles, Los Angeles, CA 90024, USA

## Abstract

Lyme Disease, or Lyme Borreliosis, caused by *Borrelia burgdorferi* and spread by ticks, is mainly known to cause arthritis and neurological disorders but can also cause psychiatric symptoms such as depression and anxiety. We present a case of a 37-year-old man with no known psychiatric history who developed panic attacks, severe depressive symptoms and suicidal ideation, and neuromuscular complaints including back spasms, joint pain, myalgias, and neuropathic pain. These symptoms began 2 years after being successfully treated for a positive Lyme test after receiving a tick bite. During inpatient psychiatric hospitalization his psychiatric and physical symptoms did not improve with antidepressant and anxiolytic treatments. The patient's panic attacks resolved after he was discharged and then, months later, treated with long-term antibiotics for suspected “chronic Lyme Disease” (CLD) despite having negative Lyme titers. He however continued to have subsyndromal depressive symptoms and chronic physical symptoms such as fatigue, myalgias, and neuropathy. We discuss the controversy surrounding the diagnosis of CLD and concerns and considerations in the treatment of suspected CLD patients with comorbid psychiatric diagnoses.

## 1. Introduction

Lyme Disease, caused by the spirochete* Borrelia burgdorferi* and transmitted to humans by ticks (*Ixodes *spp.), is reported to be one of the most common vector-borne infections in the United States [1]. In a subset of patients previously infected with Lyme Borreliosis (LB), psychiatric sequelae can occur, most commonly depression with reports ranging across studies from 26% to 66% [2]. Additionally, there are case reports of panic attacks in patients with LB infections and a reported 5.3% prevalence of panic disorder in patients at a Lyme Disorders clinic [3–6]. We present a case of a man who has no known prior psychiatric history who presented to the hospital with severe anxiety, panic, depression, and suicidal thinking in addition to multiple somatic symptoms that began 2 years after receiving antibiotic treatment for LB.

## 2. Case

The patient was a 37-year-old employed man who self-presented to the emergency department complaining of severe anxiety and suicidal ideas. For 3 months he had been experiencing palpitations, tremulousness, chest pressure, choking sensation, and an intense fear of dying. These symptoms would last for hours. He began seeing a psychiatrist 2 months prior and was started on sertraline that was titrated up to 200 mg a day, along with clonazepam 0.5 mg as needed (he was taking around 1-2 mg a day on average). The patient experienced minimal relief of his symptoms despite these therapies. He felt that the sertraline was worsening his symptoms and was tapered off of it. He reported back pain and muscle spasms, weakness and tingling in his arms and legs, and general fatigue. His physical symptoms contributed to his poor sleep, low energy, loss of interest in his job and social activities, and loss of appetite, with a 10-pound weight loss during the previous 2 months. He also reported a sense of hopelessness that “things will never get better” and was having persisting suicidal thoughts without intent or a plan. He reported no prior history of suicide attempts or self-injury. The patient stated that he had no psychiatric contact before two months. He reported that he had been diagnosed with Lyme Disease 2 years prior when he developed fatigue, tinnitus, headaches, fever, and other flu-like symptoms 1 month after pulling a tick off his leg. He had a positive Lyme ELISA antibody at that time from a local hospital laboratory. He had been treated with doxycycline 100 mg twice daily for 3 weeks. Besides some occasional mild headaches he had an almost 2-year cessation in symptoms until he began developing muscle aches, spasms, fatigue, and worsening anxiety in the past 2 months. He reported having presented to the ER multiple times for his anxiety and neuromuscular complaints but that an ELISA Lyme antibody test, from 3 weeks prior, had been negative (no Western Blot was sent since the ELISA was negative), as was the rest of his medical workup.

The patient was admitted to inpatient psychiatry for depression and suicidal ideas and treated with escitalopram which was titrated up to 10 mg a day with some improvement in mood. Nonetheless, he complained that his cramping, flank pain, and fatigue and panic did not improve. He required several doses of as-needed lorazepam which improved his anxiety symptoms only slightly. He was started on propranolol 10 mg three times daily but noted no improvement in his anxiety. Concurrently, he was seen by a Medicine consultant whose physical exam, including a complete neurological and cranial nerve examination, was unremarkable. Despite report of arthritic pain his knees and other joints were not swollen on exam. His Lyme Western Blot was negative (fewer than 5 out of 10 of the following IgG bands: 18, 23, 28, 30, 39, 41, 45, 58, 66, or 93 kDa; fewer than 2 of the following IgM bands: 23, 39, or 41 kDa), and other blood work, including a comprehensive metabolic panel, CBC, TSH, RPR, Vitamin B12, Folate, ESR, ANA, and CRP, was all negative or within normal limits (except for moderately low platelets, 120 × 10^9^/L). His urine drug screen was negative and he denied using or abusing any illicit drugs or alcohol. He denied taking any over-the-counter or other nonprescription medications. His ECG showed normal sinus rhythm. His Mini-Mental State Exam was normal (30/30) despite his complaint that he was very forgetful at home and would often not recall having taken his medications or where he had left common household items. He also complained of poor attention and focus. The patient was frustrated by the persistence of his neurological symptoms which further contributed to his hopelessness and passive thoughts of dying. He agreed to adding mirtazapine 15 mg at bedtime to his regimen and his mood symptoms improved. He however continued to complain of fatigue, weakness, back and leg spasms, and “shooting pains” in his hands, stating that the pain and tingling were “rising up” in his body. The patient, however, no longer felt unsafe and agreed with plan for discharging to home, on Day 8, with referrals for a psychiatry, neurology and internal medicine appointment.

The patient contacted the hospital's outpatient clinic 9 months after discharge to arrange reinstitution of care. He states that after discharge he visited multiple internists and neurologists and had several ELISA Lyme tests which were negative and Western Blots which were also negative for the required IgM and IgG bands (see above). He then traveled to a specialist in another state who sent blood work to IGeneX Laboratories, Inc., in California, which came back positive for Borrelia and Babesiosis on Bands 31 and 34 for IgG. In addition, he reported that his CD57 count was low (352). He was begun on a 6-month course of oral antibiotics including 3 months of tetracycline and 3 months of azithromycin and fluconazole. At that time, his brain MRI with and without contrast was negative for any white matter or other changes or disease. The patient denied further cognitive deficits since his antibiotic treatment. Patient attempted to have neuropsychological testing and functional imaging but this was not covered by his insurance and he could not obtain them due to the cost.

He stated that he was no longer taking SSRI medication or mirtazapine and only rarely took lorazepam as needed for anxiety but was eventually tapered off per the patient's request. He no longer reported panic attacks or periods of hopelessness or suicidal thoughts. He said he was trying to use relaxation and yoga and natural approaches to his anxiety and physical symptoms. Nonetheless, he reported being despondent and frustrated by the lack of improvement in his physical symptoms, which caused him to have to leave his job and go on disability. He still has fatigue, weakness, low energy, pain and spasms in his back and upper legs, and nonspecific joint pain but stated he was only on multivitamins and minerals and no other standing medications. He agreed to a low dose of gabapentin, 100 mg three times daily, which was titrated up as tolerated and helped alleviate some of his anxiety and neuropathic pain.

## 3. Discussion

Our patient presented with psychiatric symptoms and hospitalization about 2 years after a recommended course of antibiotics for diagnosed LB. His persisting psychiatric symptoms and lack of prior history of panic or depression bring up the possibility that his previous infection with LB played a role in the later development anxiety and panic attacks. There is ample evidence supporting the emergence of psychiatric symptoms including severe anxiety and depression, even months to years, after diagnosis and treatment of Lyme Disease [2–7]. The patient's laboratory studies and clinical exam did not exhibit any other signs or symptoms to suggest rheumatologic diseases like lupus and rheumatoid arthritis or a malignancy. He had no neurological sequelae and a negative brain MRI (which ruled out diseases such as multiple sclerosis) and despite his report of cognitive deficits he had no objective symptoms on admission. Unfortunately he never received neuropsychological testing or underwent SPECT or PET imaging [8]. He also was not tested for immune markers like cytokines, an important consideration given that CNS inflammation may be a part of untreated Lyme or prolonged exposure to Borrelia before initial treatment [9].

The diagnosis of “chronic Lyme Disease” (CLD) is controversial and there is conflicting evidence on whether physical symptoms can persist in Lyme after a person is treated with a recommended course of antibiotics [10]. There is evidence for a “post-Lyme Disease Syndrome” (PLDS) which may cause persisting fatigue, myalgias, and neurocognitive deficits that can remain for years even after treatment, exclusive of and distinct from fibromyalgia or chronic fatigue syndrome (CFS) [10, 11]. PLDS, however, has criteria which exclude the diagnosis if the subjective symptoms are not present within 6 months of diagnosis of Lyme or completion of antibiotic treatment or if there is a coinfection [10]. For PLDS, several randomized, controlled studies have reported improvement in neurocognition or fatigue after 6 or more months of IV antibiotic treatment although the risk of serious side effects limits this practice [10, 12]. In our case, the patient's episodic pain and fatigue are not consistent with fibromyalgia (absence of the trigger points on exam) or CFS (severe fatigue did not persist for 6 or more months) and he had no history of either prior to his first Lyme infection. The patient also had symptom onset around 2 years after his infection and no evidence of a recent reinfection due to negative Western Blot and a positive Babesiosis test, which rule out PLDS. We considered several other diagnoses including coinfections with Bartonella, Rocky Mountain spotted fever, West Nile, and other mosquito-borne illnesses but ruled them out due to the absence of laboratory or clinical evidence and the unlikelihood of persisting symptoms after recommended antibiotic treatment [13].

Many infectious disease experts refute the existence of CLD on the grounds that there is no evidence of any latent spirochetal infection that would explain the ongoing symptoms or that the person's presentation can be explained by another diagnosis like fibromyalgia, CFS, or psychiatric disorders like depression [10, 14–17]. Some Lyme experts posit ethical concerns about providing patients with hope of a potentially unidentifiable cause to their distress and then offering prolonged and potentially unsafe courses of antibiotics without clear efficacy [10, 16]. They note that advocacy groups encourage patients to seek out “Lyme-literate medical doctors” (LLMDs) and be wary of the so-called “expert” recommendations [16, 17]. Additionally, there previously had been concerns about the validity of the positive results of Lyme tests of certain laboratories including IGeneX, where our patient had a positive result, although proponents of the laboratory note that IGeneX, Inc., is licensed in several states and tests for bands not measured at other laboratories, which use CDC criteria [16, 18, 19]. Also, the utility of natural killer T cell counts (like CD57) as a marker is controversial [20].

In patients experiencing chronic fatigue, depression, and unexplained anxiety and suffering from cognitive deficits, a diagnosis and concomitant treatment hold much promise and relief for the patient and provider [21]. A recent study reported that 30% of patients presenting to a Borrelia clinic has “Organically Unexplained Symptoms” and that this group had more symptoms and worse outcomes, were more dissatisfied with their care, and felt less reassured by the medical establishment [22]. Indeed, our patient felt stigmatized and marginalized by many providers and was labeled as “psychosomatic” by some and by others led to believe that all of his symptoms were related to Lyme or related infections and was given protracted courses of antibiotics. Auwaerter and Melia, referencing Whitson and Galinsky, posit the idea that “the suggestion that an infectious agent continues to cause chronic symptoms may speak to the observed tendency for patients to develop illusory patterns of perception when they lack control” [17, 23]. Well-meaning physicians often reinforce a patient's fixation on the diagnosis by empirically prescribing antibiotics for those with nonspecific symptoms and negative or nondiagnostic Lyme serology or those with nonspecific symptoms and positive Lyme disease serology.

It is further conceivable that beyond CLD as an explanation for the patient's symptoms is the well-documented response of depression and anxiety symptoms secondary to chronic illness [24]. The patient went through a prolonged period of uncertainty, was subjected to multiple treatment therapies, and experienced new onset and distressing symptoms; this experience in its entirety could likewise exacerbate the patient's presenting symptoms. In reality, the optimal approach in cases like ours would have laid somewhere in between both a purely biological and psychological explanation and treatment frame, where this patient would have received ongoing psychiatric care, including antidepressant medications and possibly anxiolytics to treat his mood and anxiety symptoms, and psychotherapy such as cognitive behavioral therapy (CBT) to help him cope with his chronic physical illness and ongoing treatment and evaluation of his physical symptoms, with a clear explanation that a firm diagnosis may not be reached. In fact current research shows a strongly synergistic effect of both medication and CBT intervention in treating chronic illness associated with mood symptoms [25]. Treatment with pregabalin or gabapentin, as is being used in our patient, may also alleviate anxiety and underlying neuropathic pain symptoms as well, although their use in anxiety is not FDA-approved and thus is off-label [10, 26].

While we do not set out to argue the clinical validity of a diagnosis of CLD and its correlation to neuropsychiatric symptoms, our hope was to present a case that would illustrate the complexity of evaluating and treating a patient with chronic and disabling physical and psychiatric symptoms but without a clear diagnosis. In treating a patient with a history of suspected Lyme, special attention should be paid to ruling out other infections and Lyme reinfection and to diagnosing preexisting psychiatric conditions before a definitive diagnosis is made. These should be done by taking a thorough history, gathering collateral and old records, and consulting qualified specialists, with whom care should be coordinated, along with the patient's internist, to ensure the patient feels his concerns are being addressed, while minimizing the patient's tendency to overuse the healthcare system including requesting unnecessary tests or treatments. Additionally referral to therapy, specifically evidence based modalities such as CBT, can help to address and empower the patient faced with distressing psychological symptoms and a prolonged diagnostic and treatment course. Most importantly, a patient and empathic stance are crucial to ensure that patient remains connected to care and feels as if his or her concerns are being heard [10].
